# Mapping white matter tracts with SEEG electrodes

**DOI:** 10.1111/epi.70038

**Published:** 2025-11-26

**Authors:** Davide Giampiccolo, Jan van Dijk, Alejandro Granados, Fenglai Xiao, Giorgio Fiore, Roman Rodionov, Kuo Li, Aleksander Leon Lysomirski, Andrew W. McEvoy, Beate Diehl, John S. Duncan, Fahmida Chowdhury, Anna Miserocchi

**Affiliations:** ^1^ Department of Epilepsy UCL Queen Square Institute of Neurology, University College London London UK; ^2^ Victor Horsley Department of Neurosurgery National Hospital for Neurology and Neurosurgery, Queen Square London UK; ^3^ Institute of Neuroscience, Cleveland Clinic London London UK; ^4^ School of Health & Medical Sciences, City St. George's University of London London UK; ^5^ School of Biomedical Engineering and Imaging Sciences King's College London London UK; ^6^ Unit of Neurosurgery, Fondazione IRCCS Ca' Granda Ospedale Maggiore Policlinico Milan Italy; ^7^ Department of Pathophysiology and Transplantation University of Milan Milan Italy

**Keywords:** epilepsy, epilepsy surgery, SEEG, white matter anatomy

## Abstract

**Objective:**

Stereo‐electroencephalography (SEEG) is designed to record gray matter (GM) activity for epileptogenic zone localization. SEEG electrodes, however, traverse white matter (WM) pathways that connect regions involved in seizure networks and cognition. We retrospectively evaluated WM tract sampling in patients with SEEG to identify pathways suitable for prospective WM stimulation studies and validated this in two patients.

**Methods:**

This retrospective analysis included 86 individuals who underwent SEEG implantation for drug‐resistant epilepsy between 2014 and 2020. Electrode contacts were localized using postoperative high‐resolution computed tomography (CT), co‐registered to preoperative 3T T1‐weighted magnetic resonance imaging (MRI) and normalized to Montreal Neurological Institute (MNI) space. Tissue classification utilized the Harvard–Oxford atlas, whereas WM tract involvement was assessed using probabilistic WM atlases. Preoperative tractography with dissection of the optic radiation was performed and used for 50 Hz, image‐guided SEEG stimulation of white matter.

**Results:**

Among 86 patients (30 left, 40 right, 16 bilateral hemisphere implantations), 860 electrodes (6372 contacts) were implanted. Of 5853 intraparenchymal contacts (92%), 1826 (31%) were positioned exclusively within WM and 2554 (44%) at the GM/WM boundary. Patients had an average of 10 electrodes (74 contacts). Of intraparenchymal contacts, 4381 (75%) crossed WM pathways (average 21 tracts per patient). The most frequently sampled tracts were commissural fibers (corpus callosum: 100% of patients), followed by association fibers including the inferior fronto‐occipital fasciculus (97.7%), superior longitudinal fasciculus II (96.5%), and cingulum (95.4%). In two separate patients, WM stimulation induced phosphenes at .5–1 mA in different distant electrodes with contacts in the optic radiation, which was reproducible and tract selective.

**Significance:**

SEEG electrodes sample WM structures in approximately two‐thirds of contacts, and association pathways demonstrate near‐universal sampling, making them optimal candidates for systematic WM stimulation protocols. SEEG–WM stimulation with preoperative tractography was validated in two different patients.

**Key points**

- Stereo‐electroencephalography is designed primarily to record gray matter; however, electrodes traverse white matter in two‐thirds of intraparenchymal contacts.
- Long associative tracts like the superior longitudinal and inferior fronto‐occipital fasciculi are crossed in more than 95% of implantations demonstrating near‐universal sampling.
- Targeted white matter stimulation of the optic radiation is feasible with behavioral responses already at .5 mA.
- SEEG offers a unique framework for prospective white matter functional mapping in epilepsy patients.

## 1 INTRODUCTION

Epilepsy is increasingly considered a network disorder, with white matter pathways connecting the cortical and subcortical regions involved in seizure activity.^1^, ^2^, ^3^ Although it is accepted that epileptic activity spreads along white matter,^4^ thus interacting and even impairing functional brain networks, how epileptic and functional networks interact remains elusive.

From the 1950, Talairach and Bancaud^5^, ^6^ first showed that long, intracranial electrodes (stereo‐encephalography [SEEG]) inserted via stereotactic frames could be used to record epileptic activity in cortical gray matter. Although this technique was initially limited to France and Italy, since 2010 it has gained widespread adoption for invasive recording.

SEEG electrodes offer several advantages over traditional cortical grids despite some limitations in sampling lateral neocortical regions. First, they enable precise sampling of medial structures, including the amygdala, hippocampus, and medial occipital, frontal, and temporal regions. Second, they can access intrasulcal gray matter, which is critical in some pathologies such as bottom‐of‐sulcus dysplasias.^7^ Finally, they present a significantly lower risk for comorbidities including infection compared to grid implantation.

An unexplored aspect of SEEG is its potential for studying white matter function. As depth electrodes travel through the brain to reach medial structures, they necessarily cross white matter regions and therefore are ideally placed to explore their function. Direct white matter stimulation has been historically performed during awake surgery to interrogate these networks and thus avoid neurological deficits.^8^, ^9^, ^10^ However, our understanding of white matter's role in cognition remains limited because awake surgery focuses on preserving rather than characterizing these structures. Consequently, our functional knowledge of white matter pathways is biased toward language tracts, particularly the arcuate fasciculus,^9^ since awake surgery is indicated for language preservation to be performed in the so‐called dominant hemisphere.^11^

SEEG offers several unique opportunities for advancing white matter research. Its combination with preoperative tractography may allow precise targeting of tracts of interest in a hypothesis‐driven manner. It may enable systematic mapping of white matter pathways while allowing integration of multiple neurocognitive tests to identify functional overlap between traditionally separate domains, such as language and motor cognition, that may share white matter anatomy. Furthermore, stimulation in a telemetry setting may permit more sophisticated testing of complex functions such as fluid intelligence or decision‐making that cannot be assessed during awake surgery. In addition, right‐sided epilepsy means the patient would be implanted and tested in the right hemisphere, which is rarely explored during awake surgery.

In summary, SEEG white matter stimulation could provide a new approach to causally map the brain with greater resolution and breadth than current awake surgery techniques. In this work we evaluated contact locations and cortical and subcortical white matter sampling patterns in 86 individuals with epilepsy who underwent invasive recordings with SEEG for drug‐resistant epilepsy. Validated white matter atlases^12^ were used to identify tracts with the highest probability of being sampled across patients, highlighting those most suitable for prospective SEEG white matter stimulation. Finally, successful white matter stimulation of the optic radiation was performed in two patients who underwent SEEG for right drug‐resistant posterior quadrant epilepsy.

## 2 METHODS

### 2.1 Participants

Eighty‐six patients who underwent intracranial SEEG for evaluation of the underlying epileptogenic zone with invasive recordings from 2014 to 2020, at the National Hospital for Neurology and Neurosurgery, were recruited in the study. Twenty‐three of these were previously analyzed as part of a technical paper on contact localization and electrode bending.^13^ The study was conducted in accordance with the ethical standards of the Declaration of Helsinki and approved by the Health Research Authority (Ref: institutional review board [IRB]: 22/SC/0016 and 16/LO/0618).

### 2.2 Imaging acquisition

Structural magnetic resonance imaging (MRI) datasets were collected preoperatively on a 3T GE Discovery MR750. This included (i) T1‐weighted imaging that was 1 mm isovolumetric, performed with inversion‐recovery fast spoiled gradient recall echo (echo time [TE] 3.1 ms, repetition time [TR] = 7.4 ms, inversion time = 400 ms, field of view [FOV] = 224 × 256 × 256 mm, matrix = 224 × 256 × 256, parallel imaging acceleration factor = 2) and a (ii) coronal T2‐weighted sequence (TE = 30/119 ms, TR = 7600 ms, FOV = 220 × 220 mm, matrix = 512 × 512, slice thickness = 4 mm, voxel size = .43 × .43 × 4.00 mm = .74 mm^3^, SENSE factor = 2). Postoperative head computed tomography (CT) head optimized for SEEG electrodes (Siemens SOMATOM X.cite, field of view [FOV] = 512 × 512 × 332, voxel size = .43 × .43 × .50 mm^3^) was performed after implantation.

### 2.3 Imaging processing

All preoperative T1‐weighted images underwent cortical parcellation using GIF (geodesic information flow).^14^ To automatically identify SEEG location for each contact in the native space of each patient we used the algorithm developed by Granados et al.^15^ This used a combination of preoperative T1 MRI with GIF parcellation and post‐implantation CT registered to the native preoperative T1 of each patient. First, for each SEEG electrode, contact locations were extracted in the native space. Then, these coordinates were transformed into MNI space coordinates via normalization with NiftyReg. The transformation consisted in applying a rigid registration first, followed by an affine registration, and then a nonlinear (F3D) registration. The native T1 is initially skull‐stripped using SynthStrip (https://surfer.nmr.mgh.harvard.edu/docs/synthstrip/). Then the skull‐stripped T1 image in native space is used as the starting floating image and the skull‐stripped Montreal Neurological Institute (MNI) image (1 mm × 1 mm × 1 mm as implemented in FSL [https://fsl.fmrib.ox.ac.uk/fsl/docs/#/]) is used as a reference.^16^ Finally, given the three‐dimensional (3D) coordinates of SEEG contacts in MNI space, we centred a 2 voxel × 2 voxel × 1 voxel window into the location of each contact to create a binary mask image to match contact size in the electrodes used for implantation, which were either Spencer Probe electrodes (6 contacts, 2.41 mm contact length, 1.12 mm contact diameter, 1–3 5 mm contact spacing; 4–6 10 mm contact spacing) or equally‐spaced contact electrodes with reduced diameter (“Reduced diameter”) (6–10 contacts 2.29 mm contact length, .82 mm contact diameter, 5–8 mm contact spacing), (AdTech Medical, Oak Creek, WI USA).

### 2.4 Cortex‐based evaluation and atlas‐based disconnectome analysis

**FIGURE 1:**
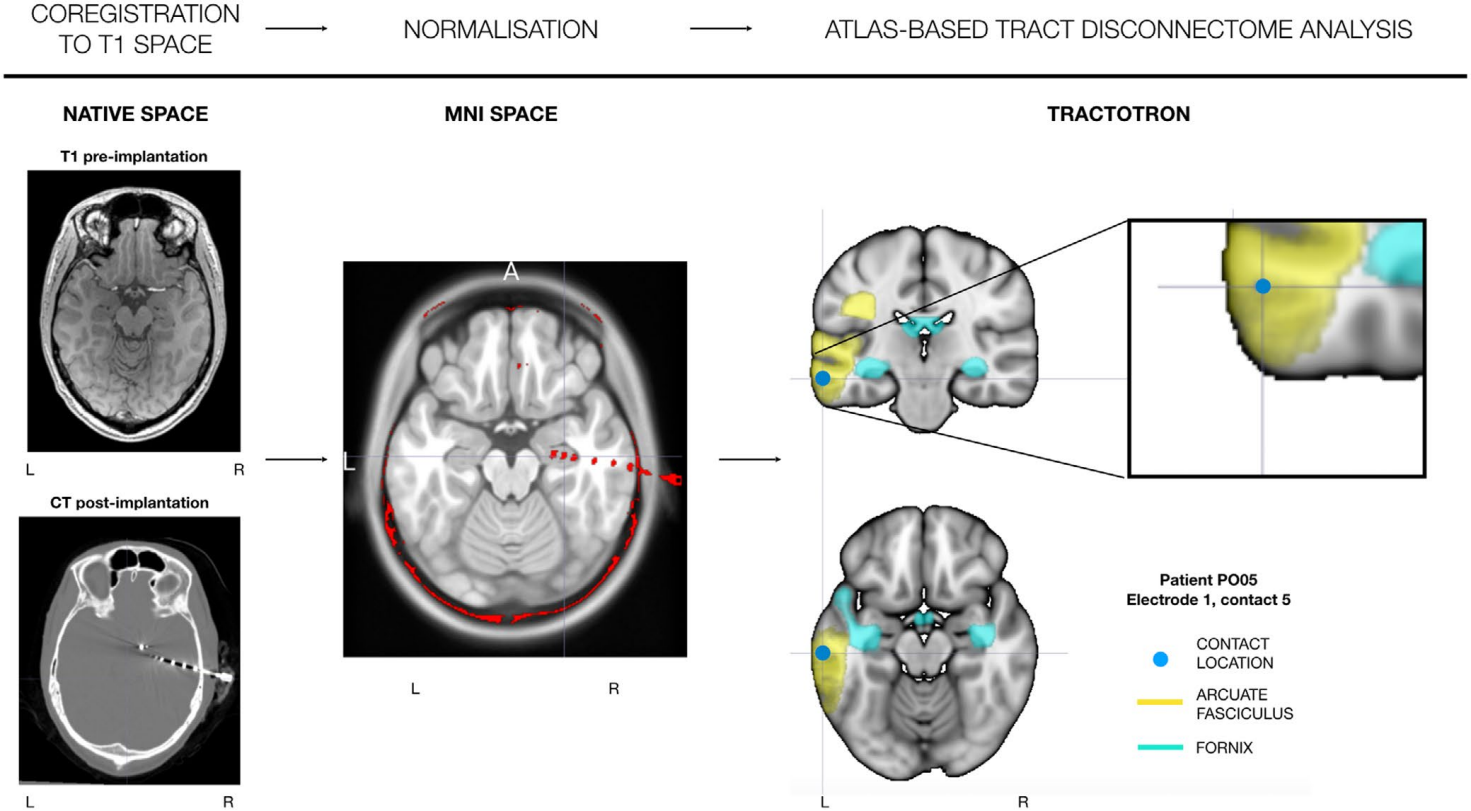
Workflow for image processing and tract crossing using Tractotron. First, preoperative T1 and post‐implantation computed tomography (CT) are co‐registered in the native T1 space. Then they are both normalized to the Montreal Neurological Institute (MNI) space. Finally, Tractotron is run on the individual contacts created by the contact coordinates to investigate which tracts are crossed by each contact in each electrode implanted.

Further assessment of cortical or white matter involvement, as well as specific tract crossing at the contact level (atlas‐based disconnectome analysis), was performed using Tractotron (BCBtoolkit; http://www.bcblab.com).^17^ To classify whether electrodes were in the cortex, gray–white matter boundary, white matter, or cerebrospinal fluid (CSF) spaces we first used a cortical vs white matter parcellation as implemented in the Oxford–Harvard cortical and subcortical atlas.^18^ A white matter atlas^12^ was then implemented in BCBtoolkit to evaluate for each contact the probability of crossing a specific white matter pathway. As each tract has a voxel value based on the proportion of a whole cohort of patients having a tract in that voxel, it can provide a probability estimation for the contact to be in the tract considering individual anatomic variability. We considered a tract as crossed if its probability of being crossed was chance or above (>50%).^1^ Finally, we evaluated the percentage of subjects having at least one contact in a tract and used this to gauge the probability for prospective patients of having at least one contact in a specific tract (Figure 1).

### 2.5 White matter stimulation with SEEG

As a validation, we included two patients who underwent SEEG–white matter stimulation of the optic radiation as part of our research setting. Details of imaging processing can be found in the Supporting Information. Briefly, individual high‐resolution diffusion imaging (multishell: b‐values: 2500, 700, 300; 101 directions, 14 b0s; 1.6 mm isovolumetric, two sets of diffusion imaging in opposite phase‐encoding) was acquired and probabilistic constrained spherical deconvolution tractography was performed with MRTrix3.^19^ Dissection of the optic radiation was performed via anatomically‐targeted automated tractography using cortical parcellations in FreeSurfer^18^ and a parcellation for the lateral geniculate nucleus from thalamus optimized multi atlas segmentation.^20^ Once the electrodes were implanted, contacts that overlapped the optic radiation and adjacent contacts were selected for stimulation using progressive current intensities from .5 to 6 mA to test stimulation effect (contacts in the optic radiation) and stimulation selectivity (contacts adjacent). Stimulation was repeated up to three times to confirm the behavioral response. Stimulation parameters are reported in detail in the Supporting Information.

### 2.6 Statistical analysis

Statistical analyses were performed in R (https://www.R‐project.org/). This included groups sorting from the database and descriptive statistics.

## 3 RESULTS

### 3.1 Contact and electrode distribution in the patients' cohort

Of the 86 patients recruited, 30 were implanted in the left hemisphere, 40 were implanted in the right hemisphere, and 16 had a bilateral implantation. There were 6372 contacts in a total of 860 electrodes. There were circa 74 ± 17 contacts per patient, for an average of 10 ± 2 electrode per patient. The average contacts used per electrode were 7 ± 2.

#### 3.1.1 Overall contact distribution

**FIGURE 2:**
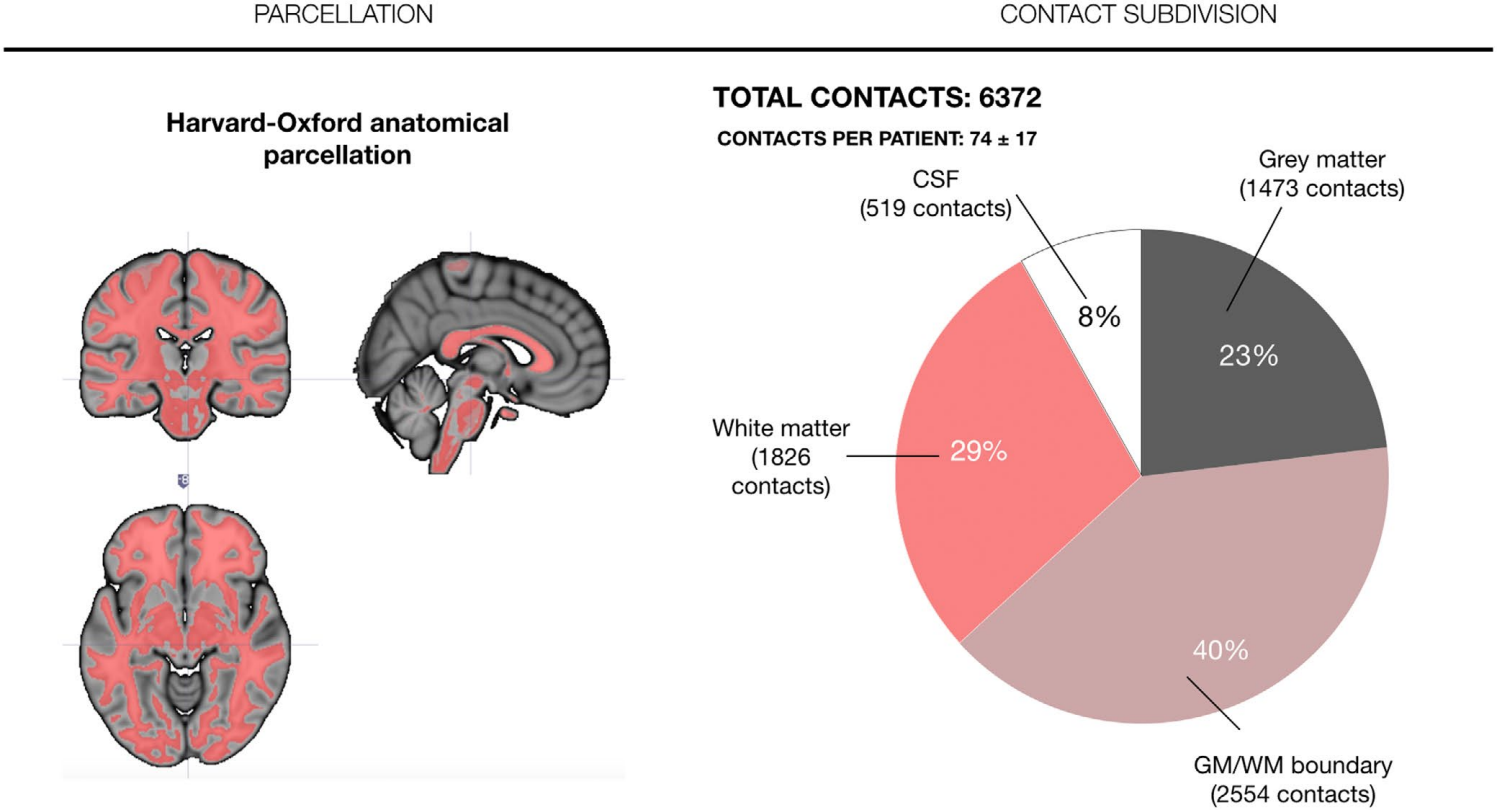
Contact subdivision according to the Harvard–Oxford tissue parcellation.

Of the total contacts evaluated, 5853 (92%) were intraparenchymal and 519 (8%) were in CSF (either ventricle or cisternae). When considering contact distribution by tissue type, 1473 contacts (25.17%) were in the gray matter, 2554 contacts (43.64%) were in the gray—white matter boundary, and 1826 contacts (31.20%) were in the white matter (Figure 2).

#### 3.1.2 Patient‐level contact distribution

Of the 74 ± 17 contacts per patient, 17.13 ± 6.56 (23% ± 7%) contacts were in the gray matter, 29.70 ± 8.44 contacts (40% ± 7%) were at gray–white matter boundary, 21.23 ± 8.10 contacts (29% ± 10.23%) were in the white matter, and 6.03 ± 6.77 contacts were in (8% ± 7.76%) CSF space.

When considering contact per electrode, of the average 7.41 ± 1.99, 1.71 ± 1.32 contacts were in the gray matter, 2.97 ± 1.63 contacts were in the gray–white matter boundary, 2.12 ± 1.85 contacts were in the white matter, and .60 ± .96 contacts were in CSF spaces.

### 3.2 Contacts crossing with white matter tracts

#### 3.2.1 Proportion of tracts crossed by SEEG contacts

Of the 5853 intraparenchymal contacts, 4381 (left: 1974 [45%]; right: 2405 [55%]) crossed white matter pathways (Table S1). Commissural fibres were the most crossed [corpus callosum: 3254 (74.28%) contacts; frontal commissure: 1767 (40.33%)] followed by frontal projection fibres [fronto‐striatal tracts: overall: 1161 (26.50%); left: 610 (13.92%); right: 551 (12.58%); anterior thalamic projections: overall 1053 (24.04%); left: 524 (11.96%); right: 529 (12.07%)]. Association fibres linking cortex to cortex within a hemisphere were the third most crossed, including the superior longitudinal fasciculi (SLF) [SLF‐I with 773 contacts (17.64%), SLF‐II with 757 contacts (17.28%), and SLF‐III with 741 contacts (16.91%)] and the inferior fronto‐occipital fasciculus was involved in 756 contacts (17.26%) (Figure S1).

Of note, when considering only contacts in white matter, 99% of contacts (1822/1826 contacts; 888 contacts in the left hemisphere; 934 in the right hemisphere) had at least two white matter tracts overlapping in one contact and 97% of contacts (1780/1826 contacts; 649 contacts in the left hemisphere; 707 in the right hemisphere) had at least three white matter tracts overlapping in a contact. This was largely caused by overlap of cortico‐cortical or projection pathways with commissural pathways, as 74% of contacts (1356/1826) showed two or more tracts overlap and 47% (865/1826) showed three or more tracts overlap in a contact when commissural fibers were excluded.

#### 3.2.2 Hemispheric differences

Few tracts showed hemispheric asymmetry in implanting. The anterior segment of arcuate fasciculus showed greater right hemisphere involvement (228 contacts, 5.20%) compared to left (84 contacts, 1.92%). Conversely, the arcuate long segment showed greater left hemisphere involvement (174 contacts, 3.97%) compared to right (85 contacts, 1.94%). The SLF‐III also demonstrated asymmetry with greater right hemisphere crossing (477 contacts, 10.89%) compared to left (264 contacts, 6.03%).

### 3.3 Individual tract probability to be crossed by at least one SEEG contact in the patients' cohort

#### 3.3.1 Patient‐level contact distribution according to individual tract

**FIGURE 3:**
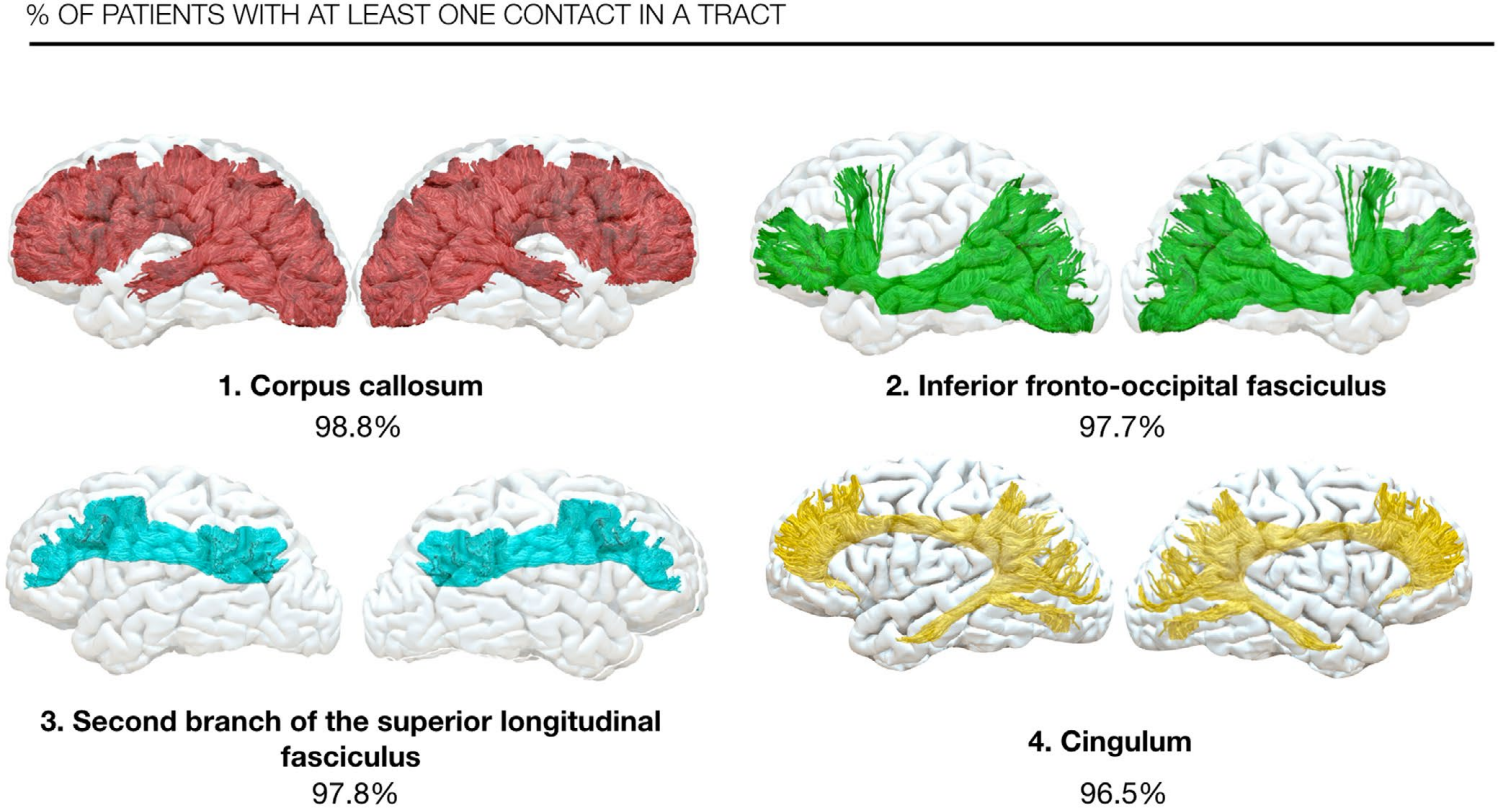
Individual tract probability to be crossed by at least one stereo‐electroencephalography (SEEG) contact in the patients' cohort for the four tracts with the highest probability.

On average, patients had 21.47 distinct tracts crossed by SEEG contacts in their implantations (Table S2). The corpus callosum was crossed in 98.85% of patients with an average of 37.40 ± 12.00 contacts per patient. Other frequently crossed tracts included the inferior fronto‐occipital fasciculus (8.69 ± 4.56 contacts per patient; 97.70% of patients), second branch of superior longitudinal fasciculus (SLF II) (8.70 ± 4.43 contacts per patient; 97.70% of patients), followed by the whole cingulum (7.41 ± 3.59 contacts per patient; 96.55% of patients) (Figure 3).

#### 3.3.2 Hemispheric differences

Hemispheric asymmetries in SEEG crossing were observed in several tracts. The anterior segment of the arcuate fasciculus was more frequently involved in the right hemisphere (83.93% of patients; 4.07 ± 3.09 contacts) compared to the left (55.56%; 1.87 ± 2.27). Conversely, the long segment of the arcuate fasciculus showed greater left hemisphere involvement (82.22%; 3.87 ± 3.19) compared to the right (57.14%; 1.52 ± 1.94). The third branch of the superior longitudinal fasciculus (SLF III) demonstrated significant asymmetry with greater right hemisphere crossing (89.29%; 8.52 ± 5.21) than the left (82.22%; 5.87 ± 4.73), in line with results proposing that the SLF‐III and the anterior segment of the arcuate fasciculus would represent the same tract.^9^, ^21^

### 3.4 Individual tract probability to be crossed at least one SEEG contact in the patients' cohort focusing on white matter contacts

#### 3.4.1 Patient‐level contact distribution in white matter tracts focusing on white matter contacts

The 1826 (29%) contacts exclusively in white matter crossed 19 distinct tracts. A similar number of tracts crossed was present also in the individual patient. The corpus callosum was crossed by white matter contacts in 100% of patients, with an average of 17.63 ± 7.17 contacts per patient, followed by the inferior fronto‐occipital fasciculus (4.73 ± 2.91 contacts per patient; 97.67% of patients), second branch of superior longitudinal fasciculus (SLF II) (4.53 ± 2.65 contacts per patient; 96.51% of patients), cingulum (3.74 ± 2.32 contacts per patient; 95.35% of patients), and fronto‐striatal tracts (6.38 ± 4.44 contacts per patient; 95.35% of patients).

#### 3.4.2 Probability of white matter tract crossing with white matter contacts

Seven tracts had white matter contact involvement in more than 90% of patients, including the corpus callosum (100%), inferior fronto‐occipital fasciculus (97.67%), superior longitudinal fasciculus II (96.51%), cingulum (95.35%), fronto‐striatal tracts (95.35%), anterior thalamic projections (94.19%), and frontal commissural fibers (93.02%) (Table 1).

**TABLE 1:** Probability of white matter tract involvement and average contacts per patient.

Tract	Overall contacts (mean ± SD)	Left contacts (mean ± SD)	Right contacts (mean ± SD)	Overall probability (%)	Left probability (%)	Right probability (%)
Corpus callosum	17.63 ± 7.17			**100.00**		
Inferior fronto‐occipital fasciculus	4.73 ± 2.91	4.66 ± 2.79	3.74 ± 2.57	**97.67**	93.18	92.59
Superior longitudinal fasciculus (SLF II)	4.53 ± 2.65	4.02 ± 2.77	3.94 ± 2.01	**96.51**	90.91	94.44
Cingulum	3.74 ± 2.32	4.41 ± 2.61	2.37 ± 1.43	**95.35**	95.45	90.74
Fronto‐striatal tracts	6.38 ± 4.44	6.07 ± 4.57	5.22 ± 3.84	**95.35**	90.91	92.59
Anterior thalamic projections	6.08 ± 4.50	5.64 ± 4.49	5.09 ± 3.89	**94.19**	93.18	90.74
Frontal commissural	8.21 ± 5.27	27		**93.02**		
Superior longitudinal fasciculus (SLF III)	4.23 ± 2.95	3.23 ± 2.96	4.11 ± 2.76	**86.05**	68.18	88.89
Frontopontine tracts	2.44 ± 2.00	2.48 ± 2.15	1.87 ± 1.57	**84.88**	84.09	79.63
Superior longitudinal fasciculus (SLF I)	3.88 ± 3.19	3.98 ± 3.31	2.94 ± 2.53	**84.88**	88.64	81.48

*Note*: Tracts are ordered according to probability of involvement. Ten tracts with the highest probability are displayed. For commissural tracts (corpus callosum, frontal commissural, fornix, anterior commissure), the same values are shown in the left and right columns, as these structures span both hemispheres. Overall probability of being involved is set in bold. Probabilities for the left and right are calculated only for patients with implantations in the respective hemisphere.

### 3.5 White matter stimulation of the optic radiation with SEEG

**FIGURE 4:**
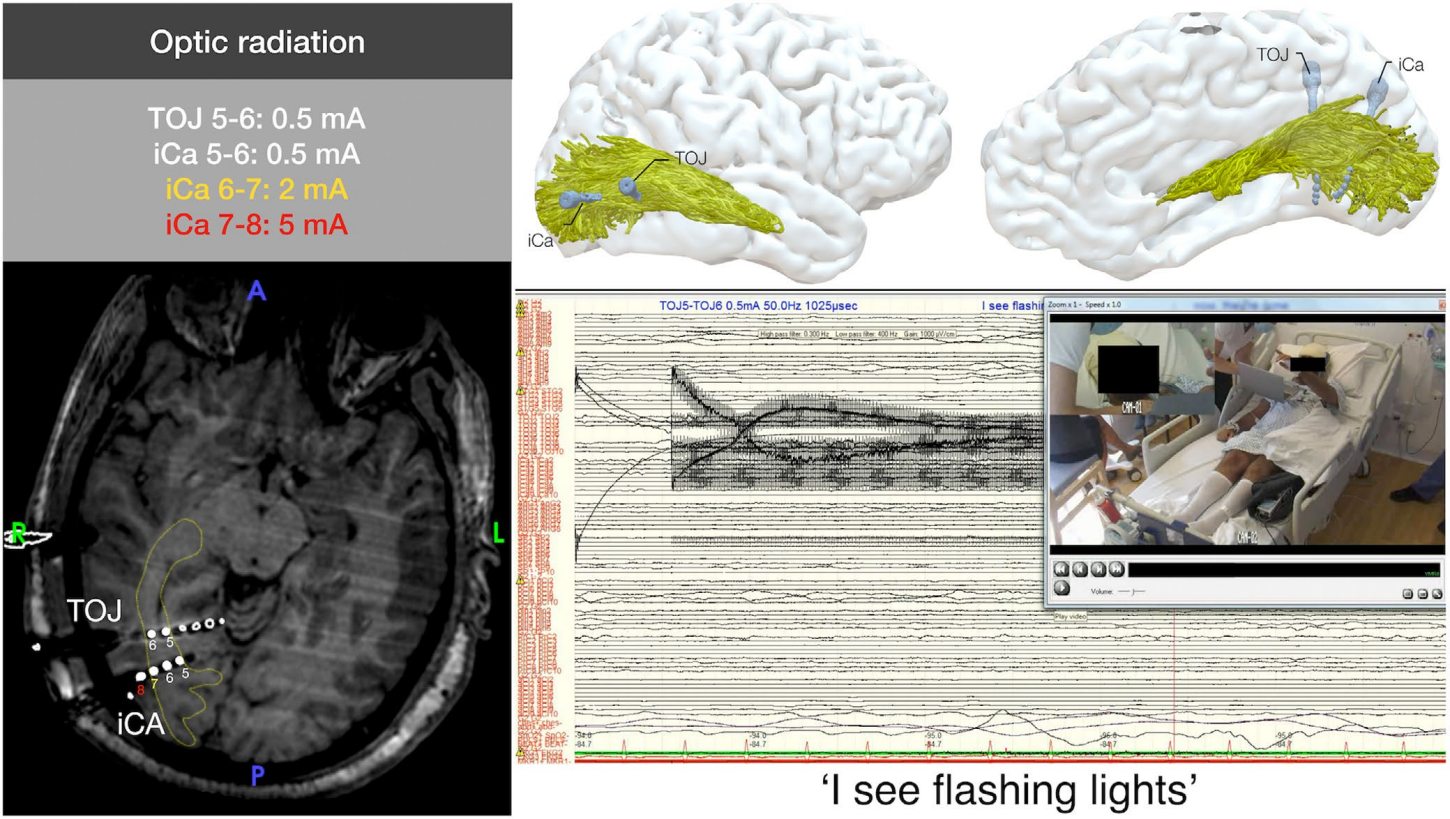
Extraoperative stimulation of the right optic radiation with SEEG in the telemetry setting. On the left, electrodes used for stimulation (TOJ, iCA) are shown in an axial plane. Contacts used by stimulation are identified with numbers. The optic radiation is shown in yellow. The different intensities required to evoke phosphenes are reported in the gray box for both electrodes. On the upper right, three‐dimensional location of the electrodes in relation to the patient's cortex and optic radiation is shown. On the lower right, a snapshot from the telemetry videorecording with invasive recordings of the patient is shown during stimulation of the TOJ 5–6 dipole (.5 mA, 50 Hz, .5 ms pulse width, biphasic, bipolar stimulation). The patient is pointing to the “flashing lights” that he sees “on the top left.”

To demonstrate the feasibility of this approach, we provide two examples of white matter stimulation mapping with SEEG in the right optic radiation. This was crossed by two different electrodes (TOJ, iCa) in a first patient with right posterior quadrant epilepsy (Figure 4, Table S3) and in one electrode (pH) in a patient with temporal lobe epilepsy. First, stimulation induced flashing lights at .5 mA in the 5–6 dipole in the TOJ electrode. These were described in the left upper quadrant, and were reported in three different trials at the same intensity. When investigating other contacts within the optic radiation in another electrode (iCa, contacts 5–6), flashing lights could also be evoked at .5 mA. It is important to note that when contrasting adjacent contacts within the same electrode (iCa 5–6; iCa 6–7; iCa 7–8), stimulation intensity required to induce phosphenes had to be increased to 2 mA in Contacts 6–7 (in which Contact 7 is at the border of the optic radiation), and further increased to 5 mA in 7–8 (in which Contact 8 was outside the optic radiation), therefore showing possible stimulation selectivity for the pathway. Similarly, the second patient had phosphenes from 1 mA stimulation of the optic radiation after stimulation, stable also at 1.5 mA and 2 mA (Figure S2, Table S4).

## 4 DISCUSSION

This study investigated the feasibility of tract‐specific white matter stimulation following SEEG implantation in patients with epilepsy undergoing invasive recordings. The analysis focused on quantifying the proportion of electrode contacts located within white matter and the probability per patient of crossing a specific white matter tract, examining various commissural, projection, and association fiber systems as defined in validated white matter atlases.^12^

Approximately two‐thirds of all contacts interfaced with white matter; 1826 contacts (29% of the total) were positioned exclusively within white matter without any contact with gray matter structures.

Finally, we examined which tracts were mostly crossed by our SEEG implantations: when considering association fibers the inferior fronto‐occipital fasciculus (4.73 ± 2.91 contacts, 97.6%), the second branch of the superior longitudinal fasciculus (SLF‐II; 4.53 ± 2.65 contacts, 96.5%), and the cingulum (3.74 ± 2.32 contacts, 95.3%). The consistent sampling of these pathways suggests they will likely be implanted in virtually all patients during future procedures, making them ideal candidates for systematic investigation during SEEG white matter stimulation protocols.

### 4.1 White matter contacts and cortical localization of function

Our data suggest that white matter contacts account for one‐ to two‐thirds of all implanted SEEG contacts. Given this substantial proportion, it is surprising that white matter stimulation has not been performed systematically to date.

Initial evidence for white matter stimulation with SEEG dates back to Talairach,^22^ who in 1965 demonstrated that clonic movement could be evoked from both projections of the primary and supplementary motor cortex. Beyond this, the literature remains largely anecdotal.^23^ A likely reason is that when a tract's activity is task dependent, stimulation alone cannot adequately probe it without concurrent behavior. This constraint has probably limited white matter stimulation with SEEG, as task‐based paradigms depend on methods that localize individual tracts before stimulation such as preoperative tractography or potentially white matter atlases. In fact, although many electrode contacts interface with white matter, we show that these are distributed across different tracts rather than concentrated in a single pathway. Because white matter function is both tract and task specific,^9^, ^10^, ^24^, ^25^ effective stimulation requires both precise knowledge of each contact's anatomic location within the white matter architecture and selection of appropriate functional tasks that engage the relevant pathways. As a result, this requires preoperative native tractography not only to identify which contacts to stimulate but also to determine suitable tasks for testing during stimulation.

In this study, we provided the feasibility of this approach by means of dissecting with tractography and stimulating the optic radiation to induce phosphenes—an established response of direct white matter stimulation of the optic radiation^26^—in two separate patients with very low stimulation intensity, .5 mA. Of note, the effect of stimulation was tract selective, as larger intensity of stimulation was required when contacts were progressively farther from the optic radiation.

Previous studies showed that recordings in the white matter of the optic radiation are possible when using SEEG in combination with visual evoked potentials (VEPs).^27^, ^28^ This suggests that together with stimulation, white matter recordings may have a role in understanding white matter contribution to cognition. However, different from the data we have provided, VEPs are not generalizable to tracts outside the optic radiation. In this context, direct SEEG stimulation of the optic radiation is a pragmatic validation target: the tract yields a well‐characterized behavioral readout (phosphenes)^26^ and the direct electrical stimulation paradigm (50–60 Hz stimulation) established in awake surgery has proven effective for interrogating multiple white matter pathways.

In summary, despite the extensive white matter sampling inherent to SEEG implantations, technical requirements may have hindered the development of systematic white matter stimulation protocols in patients with implanted SEEG. By providing preliminary data of tractography‐guided SEEG–white matter stimulation of the optic radiation, we show that anatomically‐guided, tract‐specific SEEG stimulation may represent a feasible, powerful method to map white matter tracts.

### 4.2 Targeting white matter tracts with SEEG stimulation

By leveraging white matter atlases, our data suggest that not all tracts are equally sampled by SEEG. The corpus callosum, which connects the two hemispheres, shows the highest sampling rate—an expected finding given its extensive connectivity pattern and size. Similarly, the high prevalence of multiple tracts overlap for individual contact (74%–99% of contacts had at least two overlapping tracts crossing) suggest that stimulation may cause multiple virtual disconnections rather than a specific one, as highlighted previously for awake surgery.^24^

Among lateralized tracts, long associative pathways that connect anterior and posterior regions within each hemisphere demonstrate very high sampling rates. These pathways included the inferior fronto‐occipital fasciculus, the three branches of the superior longitudinal fasciculus and the cingulum. Notably, these tracts have already been mapped during awake surgery for neuro‐oncological resections,^29^, ^30^, ^31^ thus providing established testing protocols that could be adapted for white matter SEEG stimulation during telemetry monitoring. This existing knowledge may make these tracts particularly well suited for SEEG–white matter stimulation studies, especially considering their near‐universal sampling in epilepsy patients as evidenced in this study.

Beside cognitive mapping, white matter stimulation could be potentially used for seizure induction when targeting tracts involved in the epileptogenic network. As an example, fornix stimulation has been shown to induce seizures.^32^ Although at this time, how different white matter pathways contribute to the epileptogenic network is largely unknown,^1^, ^33^ afterdischarges or even seizures generated from white matter stimulation could be potentially used in the future to delineate for each patient their individual epileptogenic network.

## 5 LIMITATIONS

To our knowledge, this is the first study specifically focused on characterizing the white matter localization of SEEG contacts. However, several methodological limitations warrant consideration when interpreting these findings.

A primary technical limitation concerns the spatial normalization approach. Contact locations were evaluated in a standardized template space rather than in each patient's native space. This methodological choice introduces potential co‐registration errors that may affect the precise categorization of contacts as being within gray matter, white matter, or CSF compartments. Small misalignments during normalization could alter the classification of contacts, particularly those positioned near tissue boundaries, potentially influencing our quantitative assessments of white matter involvement.

Another issue may be generating unwanted seizures from white matter stimulation. Although no seizures are reported in large studies for subcortical white matter mapping in awake surgery^34^ or asleep surgery,^35^ systematic mapping at stable locations may unveil white matter pathways supporting seizures that may have to be excluded for stimulation or avoided for cognitive mapping.

In addition, although this study highlights substantial white matter sampling with SEEG, it is important to acknowledge that implantation strategies vary considerably between epilepsy centers. Different institutions employ distinct planning philosophies, electrode trajectories, and sampling priorities based on their clinical experience and technical capabilities. Consequently, the proportion and distribution of white matter contacts observed in our cohort may not generalize precisely to patients implanted at other centers. These inter‐institutional differences could affect which specific white matter tracts are most consistently sampled across the broader epilepsy‐monitoring population.

Furthermore, in this study we adopted a single white matter atlas, but different white matter atlases may result in different white matter coverage.

Finally, this is a feasibility study in which mapping of an anatomically and functionally well‐described connection, the optic radiation, has been provided for causal validation in two different patients. As there is growing evidence that there is no “one tract‐one function” mapping for most white matter pathways,^36^ we believe that tract‐specific mapping with SEEG, rather than coarse, sparse whole brain mapping, should be performed to map white matter to function.^37^ Accordingly, we do not aim to characterize each tract's functional computation, but to describe the feasibility of this tract‐specific technique: tract‐specific analyses will therefore need to be performed in separate, dedicated studies. We acknowledge, however, that the limited clinical stimulation data with SEEG for tracts beyond the optic radiation remain a constraint on generalisability.

## 6 CONCLUSION

In this study, we analyzed implantation data from 86 patients who underwent SEEG for invasive monitoring of the epileptogenic zone. First, the results demonstrate that nearly two‐thirds of all electrode contacts are either positioned within white matter or directly interface with white matter structures. Second, the data identify specific white matter pathways that are consistently sampled across patients during SEEG implantation, thus suggesting the feasibility of targeted white matter tract stimulation using standard SEEG techniques. In this regard, because the inferior fronto‐occipital fasciculus, the superior longitudinal fasciculi, and the cingulum demonstrate particularly high sampling rates, these may be optimal candidates for systematic investigation through white matter stimulation protocols. In summary, this shared white matter coverage creates an opportunity for standardized approaches to assess white matter function and dysfunction (epileptogenic networks) using SEEG and preoperative native tractography or white matter atlases.

## AUTHOR CONTRIBUTIONS

Conception and design of the study: Davide Giampiccolo, John S. Duncan, Fahmida Chowdhury, and Anna Miserocchi. Acquisition and analysis of data: Davide Giampiccolo, Alejandro Granados, Giorgio Fiore, Fenglai Xiao, Roman Rodionov, Kuo Li, Aleksander Leon Lysomirski, Andrew W. McEvoy, and Beate Diehl. Manuscript draft: Davide Giampiccolo, Jan van Dijk, Beate Diehl, Alejandro Granados, Giorgio Fiore, John S. Duncan, Fahmida Chowdhury, and Anna Miserocchi.

## FUNDING INFORMATION

Davide Giampiccolo is supported by the Epilepsy Research Institute UK with an Emerging Leaders fellowship (F2403).

## CONFLICT OF INTEREST STATEMENT

The authors in this work have no conflicts of interest.

## ETHICS STATEMENT

The study was conducted in accordance with the ethical standards of the Declaration of Helsinki and approved by the Health Research Authority (Ref: IRB: 22/SC/0016 and 16/LO/0618). We confirm that we have read the Journal's position on issues involved in ethical publication and affirm that this report is consistent with those guidelines.

## Supporting information


Appendix S1.

## Data Availability

The data that support the findings of this study are available on request from the corresponding author. The data are not publicly available due to privacy or ethical restrictions.
