# Supplementary material for: Mapping white matter tracts with SEEG electrodes
Source: Epilepsia. 2025 Nov 26;67(3):1090–101. doi: 10.1111/epi.70038 (PMC13007840; doi:10.1111/epi.70038)
Supplement: Supplementary file 1 — Appendix S1. [file EPI-67-1090-s001.docx]

**Supplementary Table S1: Contact count for each tract crossed in the cohort**

| **Tract** | **Overall Count (%)** | **Left Count (%)** | **Right Count (%)** |
| --- | --- | --- | --- |
| Corpus callosum | 3254 (74.28%) |  |  |
| Frontal Commissural | 1767 (40.33%) |  |  |
| Fronto-striatal tracts | 1161 (26.50%) | 610 (13.92%) | 551 (12.58%) |
| Anterior thalamic projections | 1053 (24.04%) | 524 (11.96%) | 529 (12.07%) |
| Superior longitudinal fasciculus (SLF I) | 773 (17.64%) | 397 (9.06%) | 376 (8.58%) |
| Superior longitudinal fasciculus (SLF II) | 757 (17.28%) | 335 (7.65%) | 422 (9.63%) |
| Inferior fronto-occipital fasciculus | 756 (17.26%) | 387 (8.83%) | 369 (8.42%) |
| Superior longitudinal fasciculus (SLFIII) | 741 (16.91%) | 264 (6.03%) | 477 (10.89%) |
| Inferior longitudinal fasciculus | 672 (15.34%) | 279 (6.37%) | 393 (8.97%) |
| Cingulum | 645 (14.72%) | 354 (8.08%) | 291 (6.64%) |
| Cingulum anterior | 645 (14.72%) | 314 (7.17%) | 331 (7.56%) |
| Frontal aslant tract | 460 (10.50%) | 249 (5.68%) | 211 (4.82%) |
| Frontopontine tracts | 406 (9.27%) | 233 (5.32%) | 173 (3.95%) |
| Uncinate fasciculus | 376 (8.58%) | 210 (4.79%) | 166 (3.79%) |
| Frontal superior longitudinal fasciculus | 348 (7.94%) | 174 (3.97%) | 174 (3.97%) |
| Posterior segment of the arcuate fasciculus | 325 (7.42%) | 126 (2.88%) | 199 (4.54%) |
| Anterior segment of the arcuate fasciculus | 312 (7.12%) | 84 (1.92%) | 228 (5.20%) |
| Long segment of the arcuate fasciculus | 259 (5.91%) | 174 (3.97%) | 85 (1.94%) |
| Corticospinal tract | 247 (5.64%) | 95 (2.17%) | 152 (3.47%) |
| Frontal orbitopolar tract | 236 (5.39%) | 99 (2.26%) | 137 (3.13%) |
| Frontal inferior longitudinal tract | 148 (3.38%) | 69 (1.57%) | 79 (1.80%) |
| Frontomarginal tract | 136 (3.10%) | 43 (0.98%) | 93 (2.12%) |
| Fornix | 118 (2.69%) |  |  |
| Optic radiations | 85 (1.94%) | 42 (0.96%) | 43 (0.98%) |
| Cingulum posterior | 81 (1.85%) | 39 (0.89%) | 42 (0.96%) |
| Anterior Commissure | 69 (1.57%) |  |  |
| Hand inf u tract | 61 (1.39%) | 18 (0.41%) | 43 (0.98%) |
| Fronto insular tract 3 | 50 (1.14%) | 13 (0.30%) | 37 (0.84%) |
| Fronto insular tract 5 | 45 (1.03%) | 22 (0.50%) | 23 (0.52%) |
| Fronto insular tract 4 | 44 (1.00%) | 19 (0.43%) | 25 (0.57%) |
| Hand sup u tract | 42 (0.96%) | 19 (0.43%) | 23 (0.52%) |
| Hand mid u tract | 29 (0.66%) | 0 (0.00%) | 29 (0.66%) |
| Fronto insular tract 2 | 26 (0.59%) | 0 (0.00%) | 26 (0.59%) |
| Face u tract | 20 (0.46%) | 6 (0.14%) | 14 (0.32%) |
| Frontoinsular tract 1 | 0 (0.00%) | 0 (0.00%) | 0 (0.00%) |
| Paracentral u tract | 0 (0.00%) | 0 (0.00%) | 0 (0.00%) |

Tracts are ordered according to proportion of contacts involved. Commissural/bilateral tracts do not show lateralisation. Sup: superior; Mid: middle; inf: inferior; u tract: U-shaped fibre

**Supplementary Table S2: Proportion of tract involvement and average contacts per patient**

| **Tract** | **Overall Contacts (Mean ± SD)** | **Left Contacts (Mean ± SD)** | **Right Contacts (Mean ± SD)** | **Overall Probability (%)** | **Left Probability (%)** | **Right Probability (%)** |
| --- | --- | --- | --- | --- | --- | --- |
| Corpus callosum | 37.40 ± 12.00 | 37.40 ± 12.00 | 37.40 ± 12.00 | **98.85** | 98.85 | 98.85 |
| Frontal commissural | 20.31 ± 12.17 | 20.31 ± 12.17 | 20.31 ± 12.17 | **94.25** | 94.25 | 94.25 |
| Inferior fronto-occipital fasciculus | 8.69 ± 4.56 | 8.60 ± 4.83 | 6.59 ± 4.15 | **97.70** | 91.11 | 91.07 |
| Superior longitudinal fasciculus (SLF II) | 8.70 ± 4.43 | 7.44 ± 4.54 | 7.54 ± 4.48 | **97.70** | 93.33 | 92.86 |
| Fronto-striatal tracts | 13.34 ± 8.38 | 13.56 ± 8.64 | 9.84 ± 6.95 | **94.25** | 95.56 | 89.29 |
| Cingulum | 7.41 ± 3.59 | 7.87 ± 4.01 | 5.20 ± 2.92 | **96.55** | 93.33 | 94.64 |
| Anterior thalamic projections | 12.10 ± 8.05 | 11.64 ± 8.03 | 9.45 ± 6.95 | **93.10** | 95.56 | 87.50 |
| Superior longitudinal fasciculus (SLF III) | 8.52 ± 4.96 | 5.87 ± 4.73 | 8.52 ± 5.21 | **94.25** | 82.22 | 89.29 |
| Fornix | 1.36 ± 1.36 | 1.36 ± 1.36 | 1.36 ± 1.36 | **74.71** | 74.71 | 74.71 |
| Superior longitudinal fasciculus (SLF I) | 8.89 ± 6.59 | 8.82 ± 5.46 | 6.71 ± 6.02 | **87.36** | 91.11 | 83.93 |
| Uncinate fasciculus | 4.32 ± 2.81 | 4.67 ± 3.02 | 2.96 ± 2.22 | **90.80** | 84.44 | 82.14 |
| Frontopontine tracts | 4.67 ± 3.82 | 5.18 ± 3.25 | 3.09 ± 3.05 | **88.51** | 95.56 | 80.36 |
| Inferior longitudinal fasciculus | 7.72 ± 6.53 | 6.20 ± 4.76 | 7.02 ± 6.52 | **86.21** | 77.78 | 82.14 |
| Anterior segment of the arcuate fasciculus | 3.59 ± 2.97 | 1.87 ± 2.27 | 4.07 ± 3.09 | **79.31** | 55.56 | 83.93 |
| Frontal superior longitudinal fasciculus | 4.00 ± 3.52 | 3.87 ± 3.32 | 3.11 ± 3.27 | **75.86** | 77.78 | 67.86 |
| Long segment of the arcuate fasciculus | 2.98 ± 2.89 | 3.87 ± 3.19 | 1.52 ± 1.94 | **75.86** | 82.22 | 57.14 |
| Posterior segment of the arcuate fasciculus | 3.74 ± 3.87 | 2.80 ± 3.32 | 3.55 ± 3.90 | **66.67** | 62.22 | 62.50 |
| Frontal orbito polar tract | 2.71 ± 2.34 | 2.20 ± 1.96 | 2.45 ± 2.40 | **77.01** | 71.11 | 67.86 |
| Corticospinal tract | 2.84 ± 3.04 | 2.11 ± 2.39 | 2.71 ± 2.89 | **64.37** | 57.78 | 60.71 |
| Anterior commissure | 0.79 ± 0.97 | 0.79 ± 0.97 | 0.79 ± 0.97 | **49.43** | 49.43 | 49.43 |
| Frontal inferior longitudinal tract | 1.70 ± 2.03 | 1.53 ± 2.06 | 1.41 ± 1.91 | **55.17** | 46.67 | 48.21 |
| Optic radiations | 0.98 ± 2.08 | 0.93 ± 1.45 | 0.77 ± 1.84 | **40.23** | 44.44 | 30.36 |
| Cingulum posterior | 0.45 ± 1.26 | 0.45 ± 1.26 | 0.45 ± 1.26 | **18.39** | 18.39 | 18.39 |
| Frontomarginal tract | 0.49 ± 1.50 | 0.49 ± 1.50 | 1.07 ± 2.21 | **14.94** | 14.94 | 26.44 |
| Frontoinsular tract 3 | 0.57 ± 1.04 | 0.29 ± 0.59 | 0.66 ± 1.20 | **31.03** | 22.22 | 30.36 |
| Hand inf u tract | 0.70 ± 1.32 | 0.40 ± 0.84 | 0.77 ± 1.49 | **28.74** | 22.22 | 26.79 |
| Frontoinsular tract 4 | 0.51 ± 1.00 | 0.42 ± 0.97 | 0.45 ± 0.93 | **27.59** | 24.44 | 23.21 |
| Frontoinsular tract 5 | 0.52 ± 1.04 | 0.49 ± 1.08 | 0.41 ± 0.91 | **26.44** | 24.44 | 21.43 |
| Hand sup u tract | 0.48 ± 1.04 | 0.42 ± 1.08 | 0.41 ± 0.91 | **20.69** | 15.56 | 19.64 |
| Face u tract | 0.23 ± 0.66 | 0.13 ± 0.40 | 0.25 ± 0.74 | **13.79** | 11.11 | 12.50 |
| Fronto insular tract 2 | 0.30 ± 0.79 | 0.00 ± 0.00 | 0.46 ± 0.95 | **13.79** | 0.00 | 21.43 |
| Handmid u tract | 0.33 ± 1.00 | 0.00 ± 0.00 | 0.52 ± 1.21 | **12.64** | 0.00 | 19.64 |
| Fronto insular tract 1 | 0.00 ± 0.00 | 0.00 ± 0.00 | 0.00 ± 0.00 | **0.00** | 0.00 | 0.00 |
| Paracentral u tract | 0.00 ± 0.00 | 0.00 ± 0.00 | 0.00 ± 0.00 | **0.00** | 0.00 | 0.00 |

Tracts are ordered according to probability of involvement. For commissural tracts (corpus callosum, frontal commissural, fornix, anterior commissure), the same values are shown in left and right columns as these structures span both hemispheres. Overall probability of being involved is in bold. Probabilities for left and right are calculated only for patients with implantations in the respective hemisphere.

**
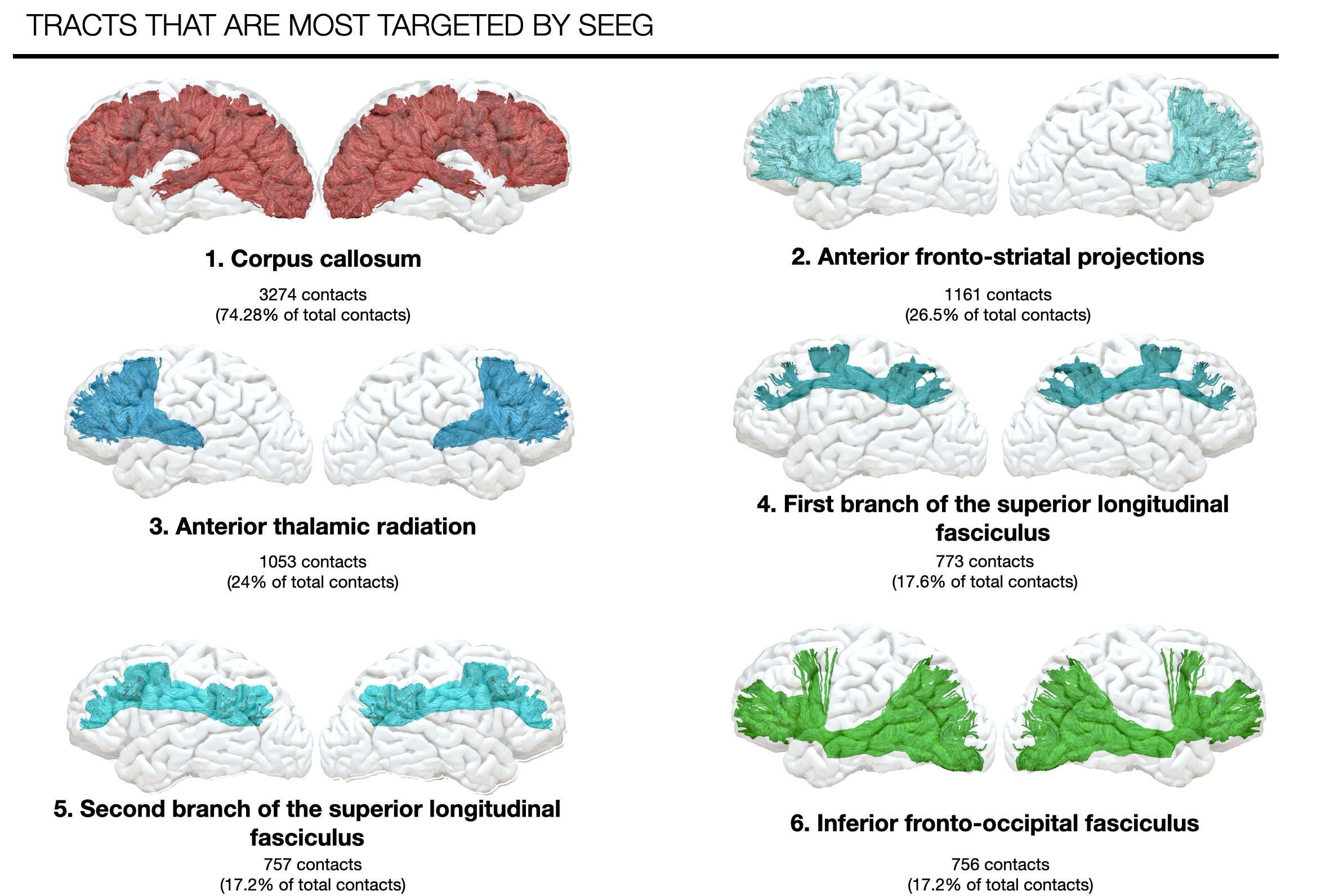
**

**Supplementary Fig. S1** Absolute number and proportion of contacts crossing the most commonly crossed tracts of the Rojkova atlas.

**Imaging acquisition and processing**

Structural MRI datasets were collected preoperatively on a 3T GE Discovery MR750. This included a (i) T1-weighted imaging was 1 mm isovolumetric, performed with inversion-recovery fast spoiled gradient recalled echo (echo time [TE] 3.1 milliseconds, repetition time [TR] = 7.4 milliseconds, inversion time = 400  milliseconds, field of view [FOV] = 224  ×  256  ×  256  mm, matrix = 224  ×  256  ×  256, parallel imaging acceleration factor = 2) and a (ii) coronal T2-weighted sequence (TE = 30/119 milliseconds, TR = 7600 milliseconds, FOV = 220 × 220 mm, matrix = 512 × 512, slice thickness = 4 mm, voxel size = 0.43 × 0.43 × 4.00 mm = 0.74 mm3, SENSE factor = 2). Diffusion MRI comprised a multi-shell acquisition (1.6 mm isotropic resolution, 101 directions, 14 b0, b-values: 300, 700, and 2500 s/mm2). Digital subtraction angiography (DSA; Siemens SOMATOM X.cite, field of view [FOV] = 512 × 512 × 383, voxel size = 0.43 × 0.43 × 0.75 mm3) was performed for vessel visualisation. A postoperative CT head optimised for SEEG electrodes (Siemens SOMATOM X.cite, field of view [FOV] = 512 × 512 × 332, voxel size = 0.43 × 0.43 × 0.50 mm3) was performed after implantation.

**Diffusion MRI pre-processing and processing**

Diffusion MRI data were corrected for noise, Gibbs ringing and signal drift using MRTrix3 (https://www.mrtrix.org).^1^ Distortion correction was performed using a synthesized b0 (Synb0-DisCo) produced from a T1-weighted MRI.^2^ The result was then included into FSL’s Topup. Magnetic susceptibility field, Eddy current and motion artifact correction were performed using FSL (<https://fsl.fmrib.ox.ac.uk/fsl>).^3^ Response functions for cerebrospinal fluid, and white and grey matter were estimated using Single-Shell 3-Tissue CSD and Multi-Shell 3-Tissue CSD in MRTrix3. Anatomically constrained tractography (ACT) using hybrid surface and volume segmentation in MRTrix3 was performed using second-order integration over fiber orientation distribution probabilistic fiber tracking algorithm selecting a maximum of 5000 streamlines from 30 million seeds.^4^ Briefly, a geodesic information flow (GIF)^5^ parcellation was used to extract cortical regions which were used as seed / termination points for tractography. Seed-based tractography using cortical terminations as seeds was performed unidirectionally twice, switching the seed and termination cortical region-of-interest (ROI). For the optic radiation, the lingual gyrus and cuneus were used as a seeding ROI, and the lateral geniculate nucleus as parcelled by THOMAS^6^ was used as termination ROI. Exclusion and inclusion ROIs were then used to remove spurious streamlines not passing via the extreme/external capsule. Fibre bundles were inspected to ensure accurate reconstruction and manual exclusion masks were used to remove spurious streamlines.

**Stimulation pipeline**

Stimulation parameters were 50 Hz, biphasic stimulation of 0.5 ms pulse width each phase, pulse duration of maximum 5 s, intensity ranging from 0.5 mA to 6 mA. Stimulation intensity was, when feasible with clinical constraints, increased in a graded fashion of 0.5-1 mA step for each task. A pair of adjacent white matter contacts in a location far from the tract of interest were used as recording reference and ground in the monopolar montage. Stimulations with after-discharges were excluded from evaluation.

**Supplementary Table. S3** Electroclinical effect of stimulation of the different contacts and the patient 1’s comments

| **TOJ 5-6** |  | | |
| --- | --- | --- | --- |
| **Trial** | **Stimulation intensity (mA)** | **Error category** |  |
| trial 1 | 0.5 | flashing lights | left side |
| trial 2 | 0.5 | flashing lights | left side |
| trial 3 | 0.5 | flashing lights | flashing lights |
| **iCa 5-6** |  | | |
| **Trial** | **Stimulation intensity (mA)** | **Error category** | **Comments from the patient** |
| trial 1 | 0.5 | phosphenes | flashing lights |
| **iCa 6-7** |  | | |
| **Trial** | **Stimulation intensity (mA)** | **Error category** | **Comments from the patient** |
| trial 1 | 0.5 |  |  |
| trial 2 | 1 |  |  |
| trial 3 | 2 | phosphenes | flashing lights on the left side |
| trial 4 | 2 | phosphenes | flashing lights on the left side |
| trial 5 | 2 | phosphenes | flashing lights on the left side |
| **iCa 7-8** |  | | |
| **Item** | **Stimulation intensity (mA)** | **Error category** | **Comments from the patient** |
| trial 1 | 0.5 |  |  |
| trial 2 | 0.5 |  |  |
| trial 3 | 0.5 |  |  |
| trial 4 | 0.5 |  |  |
| trial 5 | 0.5 |  |  |
| trial 6 | 0.5 |  |  |
| trial 7 | 1 |  |  |
| trial 8 | 1 |  |  |
| trial 9 | 1 |  |  |
| trial 10 | 1 |  |  |
| trial 11 | 1 |  |  |
| trial 12 | 2 |  |  |
| trial 13 | 3 |  |  |
| trial 14 | 4 |  |  |
| trial 15 | 5 | phosphenes | flashing lights |
| trial 16 | 6 | phosphenes | flashing lights |
| trial 17 | 6 | phosphenes | flashing lights |


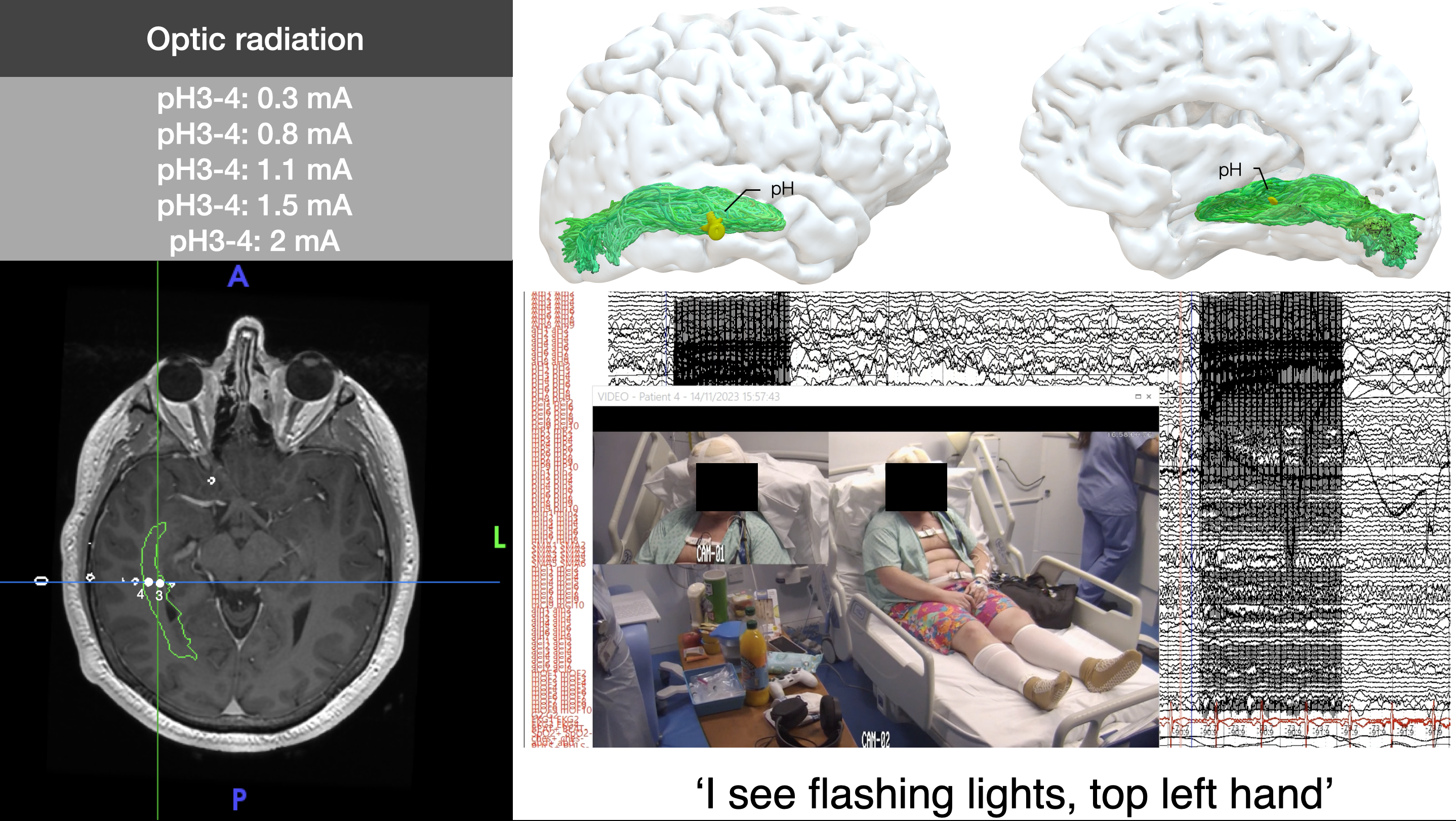


**Supplementary Fig. S2** Location of contacts in which stimulation induced flashing lights in patient 2.

**Supplementary Table. S4** Electroclinical effect of stimulation in pH and the patient 2’s comments

| **pH3-4** |  | | |
| --- | --- | --- | --- |
| **Trial** | **Stimulation intensity (mA)** | **Error category** |  |
| trial 1 | 0.3 | - | - |
| trial 2 | 0.8 | - | - |
| trial 3 | 1.1 | flashing lights | top left side |
| trial 4 | 1.5 | flashing lights | top left side |
| trial 5 | 2 | flashing lights | top left side |
| Trial 6 | 2 | flashing lights | top left side |

**Bibliography**

1. Tournier JD, Smith R, Raffelt D, Tabbara R, Dhollander T, Pietsch M, et al. MRtrix3: A fast, flexible and open software framework for medical image processing and visualisation. Vol. 202, NeuroImage. Academic Press Inc.; 2019.

2. Schilling KG, Blaber J, Huo Y, Newton A, Hansen C, Nath V, et al. Synthesized b0 for diffusion distortion correction (Synb0-DisCo). Magn Reson Imaging. 2019; 64:62–70.

3. Smith SM, Jenkinson M, Woolrich MW, Beckmann CF, Behrens TEJ, Johansen-Berg H, et al. Advances in functional and structural MR image analysis and implementation as FSL. Neuroimage. 2004; 23(SUPPL. 1):208–19.

4. Binding LP, Dasgupta D, Taylor PN, Thompson PJ, O’Keeffe AG, de Tisi J, et al. Contribution of White Matter Fiber Bundle Damage to Language Change After Surgery for Temporal Lobe Epilepsy. Neurology [Internet]. 2023; :10.1212/WNL.0000000000206862. Available from: http://www.neurology.org/lookup/doi/10.1212/WNL.0000000000206862

5. Cardoso MJ, Modat M, Wolz R, Melbourne A, Cash D, Rueckert D, et al. Geodesic information flows: spatially-variant graphs and their application to segmentation and fusion. IEEE Trans Med Imaging. 2015; 34(9):1976–88.

6. Su JH, Thomas FT, Kasoff WS, Tourdias T, Choi EY, Rutt BK, et al. Thalamus Optimized Multi Atlas Segmentation (THOMAS): fast, fully automated segmentation of thalamic nuclei from structural MRI. Neuroimage. 2019; 194:272–82.
